# Maintaining Polio-Free Status in Indonesia During the COVID-19 Pandemic

**DOI:** 10.9745/GHSP-D-21-00310

**Published:** 2022-02-28

**Authors:** Luthfi Azizatunnisa’, Utsamani Cintyamena, Vinod Bura, Asik Surya, Hariadi Wibisono, Riris Andono Ahmad, Yodi Mahendradhata

**Affiliations:** a Department of Health Behavior, Environment and Social Medicine, Faculty of Medicine, Public Health and Nursing, Universitas Gadjah Mada, Yogyakarta, Indonesia.; b Center for Tropical Medicine, Faculty of Medicine, Public Health and Nursing, Universitas Gadjah Mada, Yogyakarta, Indonesia.; c World Health Organization, Country Office for Indonesia.; d Sub-directorate of Immunization, Directorate of Surveillance and Health Quarantine, Ministry of Health of The Republic of Indonesia.; e National Certification Committee for Polio Eradication (NCCPE) Indonesia.; f Department of Biostatistics, Epidemiology and Population Health, Faculty of Medicine, Public Health and Nursing, Universitas Gadjah Mada, Yogyakarta, Indonesia.; g Department of Health Policy and Management, Faculty of Medicine, Public Health and Nursing, Universitas Gadjah Mada, Yogyakarta, Indonesia.

## Abstract

Despite the negative impact that the COVID-19 pandemic has had on polio eradication efforts, ensuring the high coverage of polio immunization and high performance of surveillance are essential to maintaining Indonesia’s polio-free status and the reaching the 2023 global polio eradication target.

## Key Messages

- Indonesia’s polio-free status as well as the 2023 global polio eradication target have been threatened by disruptions to immunization services caused by the coronavirus disease (COVID-19) pandemic and related restrictions.
- Fear of contracting COVID-19, human resource diversion, and travel restrictions posed barriers to delivering polio immunization services during the pandemic.
- To resume polio vaccination efforts and maintain polio-free status, Indonesia health authorities need to take action to:
  ∘ Maximize vaccine service reach by mapping children who have not been vaccinated and monitor the vaccine supply chain
  ∘ Integrate vaccine delivery with pandemic response efforts
  ∘ Mobilize communities for advocacy and education
  ∘ Adjust programs to address human resource, physical, and financial resource gaps
  ∘ Strengthen monitoring and evaluation and surveillance efforts

## INTRODUCTION

In the first 2 months of the coronavirus disease (COVID-19) pandemic, epidemiological evidence showed the potential magnitude of the pandemic’s indirect impacts on public health that were caused by multiple factors at various levels. Barriers to accessing health care services during the pandemic include avoidance of care, movement and transportation restrictions, social stigma, impoverishment, and the inability to pay for health services due to financial difficulties.^1^ Additionally, disruptions to medical supply chains, inadequate health care workforce, and limitations in diagnostic capacities in COVID-19 services have affected the provision of other essential health care services.^2^^,^^3^ These setbacks caused increased vulnerability to the sustainability of key health programs such as HIV, TB, and malaria; routine immunization; reproductive, maternal, newborn, child, and adolescent health; and noncommunicable diseases.^1^^,^^3^^–^^5^

The Global Polio Eradication Initiative (GPEI) has drawn global attention because of its 2023 polio eradication target.^6^ Since 1988, global polio eradication has been implemented with the commitment of all countries throughout the world. The GPEI has developed a robust global network and disease surveillance system equipped with modern laboratories and trained personnel who can collect, analyze, and disseminate information on infectious diseases. Since GPEI’s initiation, polio has become a priority in Indonesia with full support from the government. With GPEI’s assistance, Indonesia has built the capacity for effective polio outbreak response, including the ability to conduct field, virological, and epidemiological investigations and have an acute flaccid paralysis (AFP) surveillance system.^7^ The Southeast Asia region, including Indonesia, has been certified as polio-free since 2014. Currently, every country in the world has been polio-free except Afghanistan and Pakistan. With the eradication target nearly reached, GPEI developed and accelerated a transition plan to institutionalize the polio legacy into the public health system. Then, just 3 years before the meeting eradication target deadline, the COVID-19 pandemic struck.^8^

The COVID-19 pandemic affected polio eradication efforts in Indonesia in several ways. In the early months of the pandemic, GPEI recommended postponing the polio vaccination campaign until the second half of 2020 since most of the polio resources and laboratories had been shifted for the pandemic response.^6^^,^^9^^,^^10^ Due to this postponement, approximately 80 million children around the world likely missed their vaccinations.^10^^–^^12^ Even before the pandemic, Indonesia still had areas with low immunization coverage.

One of the criteria for polio-free certification is the absence of wild poliovirus (WPV) isolated from AFP cases, healthy individuals, or environmental samples. AFP surveillance and environmental surveillance are critical to monitor the circulation of WPV and vaccine-derived poliovirus (VDPV).^10^^,^^13^ Diversion of resources due to COVID-19 has arguably affected the AFP surveillance.

In addition to Indonesia dealing with pandemic response efforts, they also face the risks of importation of WPV from polio-endemic countries, emergence of circulated VDPV, and accidental release of poliovirus from laboratories and vaccine production facilities.^14^ In 2020, Indonesia had just contained a circulated VDPV outbreak in Yahukimo, Papua.^15^

Ensuring the high coverage of immunization and high performance of surveillance are essential to maintaining the country’s polio-free status. However, the negative impact of the pandemic on the polio eradication efforts has been unavoidable. We aim to describe the impact of the pandemic on the polio eradication efforts in Indonesia and the strategies to maintain the polio-free status during the COVID-19 pandemic.

## CHALLENGES/BARRIERS IN MAINTAINING POLIO-FREE STATUS

**FIGURE 1:**
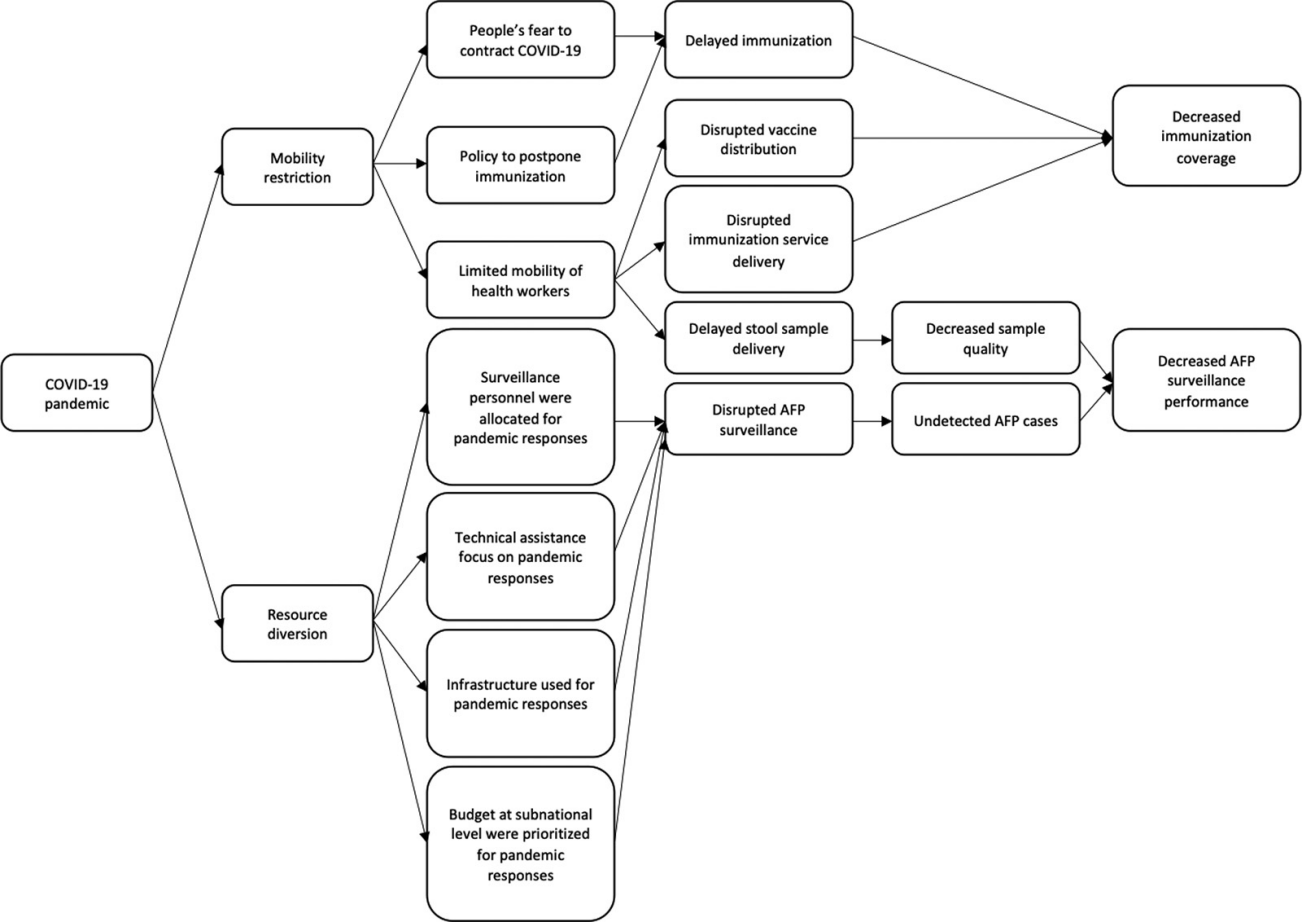
Problem Identification of the COVID-19 Pandemic’s Impact on the Polio Eradication Initiative in Indonesia Abbreviation: AFP, acute flaccid paralysis; COVID-19, coronavirus disease.

We identified both supply- and demand-side barriers to maintaining high polio immunization coverage during the COVID-19 pandemic (Figure 1).^11^^,^^12^^,^^16^

We identified both supply- and demand-side barriers to maintaining high polio immunization coverage during the COVID-19 pandemic.

### Supply Side

#### Restrictions Disrupt Vaccine Delivery

After the first case of COVID-19 in Indonesia was announced in March 2020, the government took several measures to control its spread, including recommending physical distancing, imposing restrictions on mass gatherings and travel, and banning religious congregations.^16^ The physical distancing policy led to the temporary closure or suspension of *posyandus (*integrated health posts) and *puskesmas (*primary health care centers), which are the frontline and backbone in immunization delivery, respectively. A survey conducted on immunization delivery during the pandemic showed that among the total 5,329 puskesmas that participated, 84% reported that immunization delivery was significantly disrupted due to the COVID-19 pandemic.^16^ More than 56% reported that the disruption occurred both at puskesmas and posyandu levels.^16^ Further, a study in Indonesia shows that vaccine delivery was disrupted due to mobility restriction.^17^ The polio immunization coverage in 2020 was 7% lower than in 2019 (94.2%).^18^^,^^19^ If immunization services continue to be disrupted, there is a potential risk of vaccine-preventable disease outbreaks.^16^

The travel restrictions may have been a blessing in disguise for polio eradication efforts globally because they reduced the chance of importation. However, the continued impact of travel restrictions will affect immunization coverage, which then opens the door for potential importation and possible outbreak.

Because of the disruption to immunization delivery, there is a risk of increased damaged or expired vaccines. Routinely monitoring vaccine stock to check the expiration and vaccine vial monitor status, including cold chain, temperature, and storage capacity, particularly when COVID-19 vaccines have displaced other vaccines, is essential to prepare for immunization service resumption.

#### Resource Diversion

The increasing demand for human resources in the surge condition during the pandemic could not be fulfilled by the available health care workforce. Therefore, the available human resources, including vaccinators and other health care workers, were diverted to cope with the pandemic mitigation efforts.^17^^,^^20^^,^^21^ Resources in surveillance, diagnostic laboratories, and personal protective equipment were prioritized for the nationwide emergency response to COVID-19. A study conducted in Jakarta reported that the increased number of health workers who contracted COVID-19 disrupted immunization due to resource constraints.^17^

To cope with budget constraints due to the pandemic response, the Indonesian government reallocated the national and subnational health operational budgets to be used for the pandemic responses.^22^^,^^23^ The reallocated budget was the health operational budget that was originally intended for mass-gathering activities such as workshops and posyandu.^7^ A study reported that this reallocation was implemented at all puskesmas in East Lombok District, West Nusa Tenggara Province. In puskesmas in Selong East Lombok, before the reallocation, the budget for essential services was 64% of the total puskesmas budget per year. During the pandemic, 35% of the annual budget was allocated for COVID-19 response, and the budget for essential services decreased to 40%.^24^ The budget reallocation resulted in disrupted polio vaccine delivery and decreased vaccine-preventable disease surveillance.^25^^–^^27^

### Demand Side

Vaccine hesitancy, due to religious belief, myth, and misinformation, has been a classic challenge in immunization programs. During the COVID-19 pandemic, fear of contracting the virus caused hesitancy to get the vaccine at health care facilities.^11^^,^^12^ In July 2020, the Ministry of Health, with technical assistance from UNICEF, conducted an online survey in Indonesia among parents and caregivers of children aged younger than 2 years that showed that about two-thirds (64%) of parents and caregivers sought immunizations during the pandemic, 23% refused to bring their children to get immunizations, and the remaining 13% were hesitant.^28^ These results indicated that parents and caregivers attitudes regarding immunization services had changed compared to before the pandemic despite immunization resumption in the second half of 2020.^29^

Online survey results indicated that parents and caregivers attitudes regarding immunization services had changed compared to before the pandemic.

### Impact of Pandemic on AFP Surveillance

**FIGURE 2:**
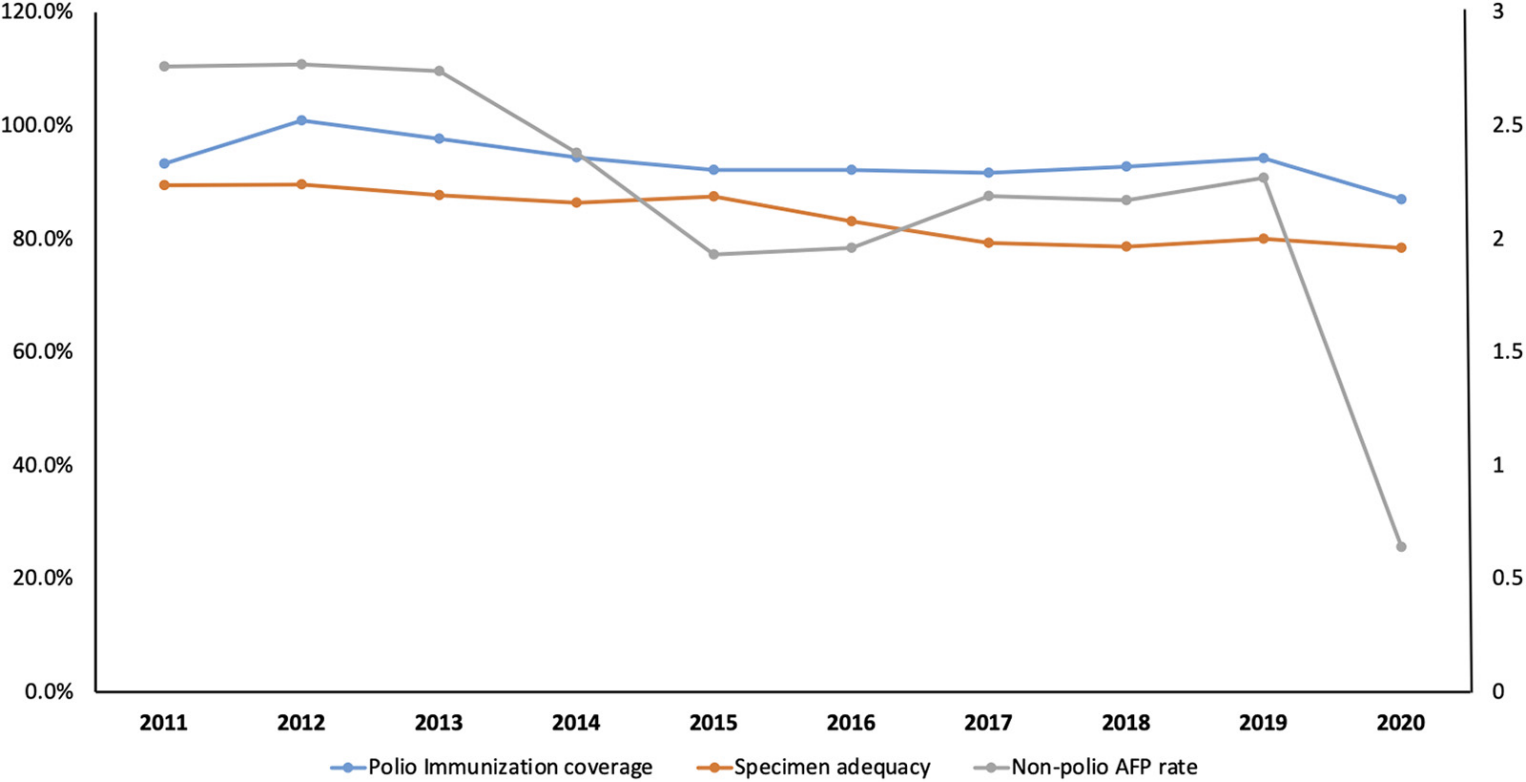
Trends in Polio Immunization Coverage, Non-Polio AFP Rate,^a^ and AFP Specimen Adequacy in Indonesia, 2011–2020^18^^,^^19^^,^^31^^–^^37^ Abbreviation: AFP, acute flaccid paralysis. ^a^2/100,000 children aged younger than 15 years.

At the beginning of the COVID-19 pandemic, AFP surveillance was suspended for safety reasons. The restrictions limited the number of stool specimens that could be collected and prolonged the delivery time.^30^ However, there was not much difference in the specimen adequacy before the pandemic in 2019 (80%) and during the pandemic in 2020 (78.4%). Restrictions also resulted in the inability to ship the stool samples and environmental samples to the World Health Organization-accredited laboratories and have them tested. As a result, the reported AFP (Figure 1) decreased.^30^ In 2020, Indonesia’s non-polio AFP rate was only one-fourth (0.64/100,000) of 2019 (2.27/100,000), which was far below the target for endemic countries (2/100,000) (Figure 2).^18^^,^^19^ This indicates that AFP surveillance has been severely disrupted during the pandemic.

Over the last 10 years, polio immunization coverage, non-polio AFP rate, and specimen adequacy reached the lowest rates in 2020 during the pandemic (Figure 2).^18^^,^^19^^,^^29^^–^^35^

## DISCUSSION

Disruption and postponement of routine immunization may escalate the risk of future outbreaks of vaccine-preventable diseases, including polio, regardless of the COVID-19 pandemic’s trajectory. Postponing polio immunization may not have a direct impact immediately, but in the long term, it can undermine the progress gained since 1988 toward polio eradication.^9^

We suggest the following recommendations to continue polio eradication efforts during the COVID-19 pandemic: (1) incorporate polio eradication efforts into the pandemic response to resume vaccine services; (2) ensure that relevant, reliable, and timely data continue to be collected on vaccine coverage rates and vaccine stock so that disruptions in services and the supply chain can be prepared for and mitigated efficiently; (3) adjust program plans accordingly to address human, physical, and financial resource gaps; (4) mobilize the community in vaccine advocacy, education, and awareness; and (5) use community-based surveillance strategies to improve AFP surveillance.

### Incorporate Polio Eradication Efforts Into the Pandemic Response

To improve vaccine coverage, the government and key stakeholders should work to resume vaccination programs quickly by establishing a committee to develop a resumption plan, monitor unvaccinated children, and identify long-term strategic adjustments for the polio eradication program. Polio eradication efforts should coexist and be incorporated into pandemic responses.^8^ National-level efforts should provide guidelines on how to resume services. In the middle of 2020, health authorities of Indonesia published technical guidance on conducting immunization services while following health protocols during the pandemic.^29^ All health workers who are involved with vaccination resumption efforts should be trained in modifying immunization delivery and following the COVID-19 preventive measures and infection control precautions (e.g., adequate personal protective equipment, safe handling of injection waste, designated waiting areas, and patient flow maps and ensuring the safety of the public).^12^^,^^38^^,^^39^ The provincial and local levels should be empowered for operational decision making based on their specific context.^8^

Polio eradication efforts should coexist and be incorporated into pandemic responses.

### Ensure the Collection of Data on Vaccine Coverage and Stock

A significant number of children have been reported to have missed their immunization.^10^^–^^12^^,^^16^ Data are crucial to ensure that children receive their vaccinations. The health authority should start to collect and analyze data on routine immunization and map the missing children. Relevant, reliable, and timely data will help to shape immunization programs and guide decisions during a pandemic,^12^^,^^39^ and to plan for programs once the pandemic subsides.^27^ The accurate data will also assist in calculating and preparing logistics and vaccines. During the suspension, the health authority should routinely monitor the vaccine stock and the vaccine vial monitor. Using data and information systems will facilitate efforts in predicting the impact of the pandemic on immunization programs and planning strategies to minimize the disruption of immunization services during the COVID-19 pandemic and beyond.

### Adjust Programs to Address Resource Gaps

At the program management levels, managers should adjust and update national and local plans to identify and develop appropriate strategic plans to address resource gaps.^40^ This includes making action plans for the full-scale campaign and supplementary immunization once the conditions allow. Context-specific guidelines will also be needed to support health care workers in delivering immunization services safely. Supervision and monitoring of adherence to implementation of the guidelines as well as to infection prevention and control measures are pivotal in this adaptive immunization delivery.^12^ Though these adjustments and modifications are needed, they pose an added challenge of potentially increasing operational costs of the immunization campaign by 36%–131%.^41^ Therefore, preparing and budgeting for financial resources for this modification should also be done.

Adjustment in vaccine stock and storage should be done to address the possible increased vaccine wastage because of the decreased number of children being immunized. Scheduling can be a solution, but health authorities should reinforce the multidose vial policy as well. Any eligible child should be encouraged to get vaccinated as soon as possible to reduce missed opportunities.^38^

Resources for mitigating the outbreak should be planned and developed since there remains a risk of a polio outbreak because of low immunization coverage caused by immunization delay during the pandemic. The likelihood of cVDPV2 occurring is high since it is the most common type (90%) of polio that occurs globally. Therefore, the health authority can prepare for a potential outbreak response by providing a monovalent type 2 oral polio vaccine, strengthening routine immunization with inactivated polio vaccine, and accelerating the availability of type 2 novel oral polio vaccine.^8^

### Mobilize the Community to Increase Advocacy and Awareness

The success of immunization campaigns depends on effective community mobilization. Ensuring the continuation of routine immunization requires involvement from all sectors at all levels.^42^ Collaboration among government, nongovernment organizations, and the private sector is pivotal in developing strategies and tools to advocate with policy makers, educate health care workers, and inform health champions and the public that immunization is essential and should be continued despite the ongoing pandemic. Strong and continuous advocacy must be conducted to maintain the prioritization of immunization as an essential health service.

Ensuring the continuation of routine immunization requires involvement from all sectors at all levels.

Community health education on the importance of immunizations must be strengthened along with COVID-19 prevention education, including motivating parents, scheduling, having a protocol to get the vaccination, and raising awareness about the adverse events.^11^^,^^39^ Use of popular and culturally acceptable media and strategies is essential in raising community awareness.^12^^,^^39^ Engaging key actors and champions who have influence in the community, such as religious leaders, community leaders, and community health workers (volunteer cadres), is also critical.^12^^,^^40^^,^^41^ Community mobilization has had a significant role in the success of polio campaigns in the past during Indonesia’s national immunization days and in other settings.^42^ This strategy can be adopted to identify the missing children during the pandemic by involving every sector related to immunization programs. Women play an essential role in social mobilization, especially at the household and community levels. Singhal identified personal rapport, credibility, and trust as the keys to successful community mobilization.^43^

### Use Community-Based Surveillance Strategies

Several studies proposed conducting AFP surveillance along with COVID-19 surveillance. Since the polio legacy in surveillance has been used in COVID-19 surveillance, the surveillance system could perform the COVID-19 surveillance while maintaining AFP surveillance. These 2 surveillance systems along with vaccine-preventable disease surveillance can be aligned to achieve each target and goal.^27^^,^^40^ However, since conducting both surveillance systems at the same time is challenging, community-based surveillance can be strengthened to maintain AFP surveillance.

Community-based surveillance has been identified as a significant effort to enhance local efforts in surveillance.^42^ In Indonesia, the pandemic has formed solidarity among community members who have cared for each other during this difficult time. Community groups have developed many initiatives to strengthen their resilience during the pandemic. This foundation is an opportunity to strengthen community-based AFP surveillance if given adequate education on polio and AFP.

Community-based surveillance has been identified as a significant effort to enhance local efforts in surveillance.

Due to the restrictions, health authorities should prepare protocols for scenarios when stool sample shipment will not be possible.^44^ Mapping each facility’s capacity to store the specimens and each laboratory’s testing capacity is needed.^44^

There is a possibility to integrate environmental polio surveillance and severe acute respiratory syndrome coronavirus 2 (SARS-CoV-2) detection through wastewater sampling. SARS-CoV-2 was found in the stool of infected persons. Several studies revealed that wastewater sampling showed the presence of the SARS-CoV-2 in the environment before COVID-19 was detected.^29^^–^35^,^^45^^–^^47^ This presents an opportunity to adapt wastewater sampling to be used as an epidemiological tool for SARS-CoV-2 detection and can be integrated with poliovirus environmental surveillance.^48^ However, this surveillance method has not yet been adopted in SARS-CoV-2 surveillance. This topic opens the opportunity for further research.

## CONCLUSIONS

To maintain Indonesia’s polio-free status, immunization coverage and performance of AFP surveillance must be kept high. Disruption due to the pandemic must be mitigated with strong policy initiatives and integration in program implementation. Modifying service delivery, mobilizing the community, adjusting program planning, and strengthening monitoring and evaluation efforts must be implemented to resume immunization delivery.

## Notes

**Peer Reviewed**

**First published online:** February 3, 2022.
